# Microbial Community-Driven Etiopathogenesis of Peri-Implantitis

**DOI:** 10.1177/0022034520949851

**Published:** 2020-08-12

**Authors:** G.N. Belibasakis, D. Manoil

**Affiliations:** 1 Division of Oral Diseases, Department of Dental Medicine, Karolinska Institute, Huddinge, Sweden

**Keywords:** peri-implant infection(s), microbiology, microbial ecology, implant dentistry/implantology, inflammation, periodontal disease(s)/periodontitis

## Abstract

Osseointegrated dental implants are a revolutionary tool in the armament of reconstructive dentistry, employed to replace missing teeth and restore masticatory, occlusal, and esthetic functions. Like natural teeth, the orally exposed part of dental implants offers a pristine nonshedding surface for salivary pellicle-mediated microbial adhesion and biofilm formation. In early colonization stages, these bacterial communities closely resemble those of healthy periodontal sites, with lower diversity. Because the peri-implant tissues are more susceptible to endogenous oral infections, understanding of the ecological triggers that underpin the microbial pathogenesis of peri-implantitis is central to developing improved prevention, diagnosis, and therapeutic strategies. The advent of next-generation sequencing (NGS) technologies, notably applied to 16S ribosomal RNA gene amplicons, has enabled the comprehensive taxonomic characterization of peri-implant bacterial communities in health and disease, revealing a differentially abundant microbiota between these 2 states, or with periodontitis. With that, the peri-implant niche is highlighted as a distinct ecosystem that shapes its individual resident microbial community. Shifts from health to disease include an increase in diversity and a gradual depletion of commensals, along with an enrichment of classical and emerging periodontal pathogens. Metatranscriptomic profiling revealed similarities in the virulence characteristics of microbial communities from peri-implantitis and periodontitis, nonetheless with some distinctive pathways and interbacterial networks. Deeper functional assessment of the physiology and virulence of the well-characterized microbial communities of the peri-implant niche will elucidate further the etiopathogenic mechanisms and drivers of the disease.

## Clinical Definition and Epidemiology of Peri-Implant Infections

Along with the advent of dental implants as a reconstructive treatment option in dentistry, peri-implant infections have emerged as a by-product of this advancement in bioengineering. Peri-implant infections are categorized as either peri-implant mucositis, if the induced inflammation is limited to peri-implant soft tissues, or peri-implantitis, if the inflammation extends to the underlying bone, further causing osteolysis. Diagnostic criteria for peri-implant infections primarily rely on clinical and radiographic examinations. Accordingly, the clinical sign of bleeding on probing (BOP) is central to detecting peri-implant inflammation in the form of mucositis. The diagnosis of peri-implantitis is commensurate with radiographic changes in crestal bone levels, particularly characterized by a symmetrical “saucer-shaped” bone defect around the implant. The latest case definitions for peri-implant mucositis include BOP or suppuration but no radiographic crestal bone loss beyond the initial remodeling. Peri-implantitis also includes further bone loss and increased probing pocket depth (PPD), compared to previous examinations (Berglundh et al. 2018). Overall, approximately one-third of all patients and one-fifth of all implants will experience peri-implantitis (Kordbacheh Changi et al. 2019). The primary risk factors coupled to these epidemiological observations are ill-fitting or ill-designed fixed and cement-retained restorations, as well as a history of periodontitis (Kordbacheh Changi et al. 2019). Smoking is also an important risk factor that is shared with periodontitis, particularly in combination with poor oral hygiene (Kumar 2019).

## Histological Particularities of Peri-Implant Sites

Manufactured primarily out of titanium, dental implants consist of an endosseous rough-surfaced part that promotes osseointegration and a transmucosal smooth-surfaced part exposed to the intraoral environment. Since they are expected to compensate for the absence of natural teeth and their physiological functions, there is also a tendency to perceive peri-implant infections as pathologies analogous to gingivitis and periodontitis of natural teeth. Nonetheless, fundamental histological and immunophysiological differences with natural teeth render dental implants more susceptible to endogenous oral infections (Belibasakis 2014; Belibasakis et al. 2015). First, whereas natural teeth are socketed into the alveolus via the periodontal ligament (PDL), osseointegrated implants are directly anchored to the bone. The resulting lack of PDL limits the blood supply to supraperiosteal vessels, thereby restricting the amount of nutrients and immune cells that may extravasate to tackle the early stages of bacterial infection. Second, fibers of the supracrestal connective tissues are positioned circumferentially around implants, not perpendicularly as into natural teeth. This anatomical-functional organization reduces the physical barrier against bacterial invasion into the submucosa and places peri-implant tissues in an “open wound” conformation.

## Ecological Characteristics of the Peri-Implant Niche

Upon implant insertion, a salivary pellicle rapidly adsorbs onto the orally exposed surfaces, which promotes the adhesion of early bacterial colonizers, in turn providing the surface receptors for the incremental coadhesion of late colonizers (Kolenbrander et al. 2010). Pellicles formed onto titanium or tooth enamel were shown to display molecular differences (Edgerton et al. 1996). Titanium pellicles formed in vitro were shown to comprise proline-rich proteins, secretory IgA, α-amylase, and high molecular weight mucins, yet lacked low molecular weight mucins and cystatins as commonly detected on enamel (Edgerton et al. 1996). Despite these potential differences, the composition of titanium-formed pellicles does not seem to influence initial bacterial adhesion (Fröjd et al. 2011). Bacterial colonization is already observed within 30 min after implant insertion and further evolves toward the establishment of organized biofilm communities in the peri-implant crevice in the next 2 wk (Belibasakis et al. 2015). In the early months following implant insertion, peri-implant biofilms were shown to display only a few differences in their taxonomic composition, yet harbored a less diverse microbiota than that of neighboring teeth (Payne et al. 2017). At this stage, the bacterial communities that colonize the peri-implant niche may reach a symbiotic equilibrium with the host and be compatible with peri-implant health. Nonetheless, factors that promote biofilm growth also favor the initiation of tissue inflammation and alter the microenvironment of the peri-implant sulcus (Belibasakis 2014). The resulting modifications in the microenvironment in turn cause dysbiotic shifts in the microbiota that exacerbate inflammatory progression and ultimately peri-implant health and implant functionality (Heitz-Mayfield et al. 2015). For instance, discontinuation of oral hygiene for a period of 3 wk was shown to increase the abundance of putative pathogens, such as *Tannerella, Prevotella, Fretibacterium*, or *Treponema* spp., that further correlated with a regional increase in proinflammatory cytokines (Schincaglia et al. 2017).

In essence, peri-implantitis is an endogenous mixed infection, occasionally implicating nontypical oral bacteria. This may imply an extraoral contribution to the infection, although it remains difficult to draw a hard line on this definition. Regardless, peri-implantitis remains a biofilm-induced condition of already osseointegrated implants (late failure), in contrast to inefficient osseointegration due to contamination during insertion (early failure) or compromised bone regeneration/reosseointegration during the surgical reconstructive phase of its treatment.

Whereas the patient’s systemic conditions or genetic susceptibility may also increase the odds for the development of peri-implant infections (Kumar 2019), the peri-implant microbiota constitutes the etiologic factor that can be more feasibly and predictably targeted by therapeutic intervention and thus requires our utmost focus and understanding.

## Implant Surface as a Modifier of the Peri-Implant Niche

The implant surface structure and abutment interface may affect microbial colonization and disease progression (Belibasakis et al. 2015; Lauritano et al. 2020), whereas modification of its characteristics may enhance antimicrobial properties and clinical outcomes (Asensio et al. 2019). The occurring biocorrosion of the implant surface can result in release of titanium particles and biological implant complications (Mombelli et al. 2018). Implant corrosion and wear may be caused by prolonged exposure of the metal surface to the biofilm or physiological friction at the implant-abutment interface. The released titanium ions and micro- or nanoparticles may affect the surrounding tissues (Apaza-Bedoya et al. 2017) and potentiate the inflammatory response of macrophages (Pettersson et al. 2017). Whether this is clinically significant for the progression of peri-implantitis remains to be proven, albeit titanium may act as priming agent of the immune response, with the microbial component being necessary for instigating inflammation. The implant material has also gained interest as a modifier of the microbial colonization (Al-Ahmad et al. 2013). While most studies on implant colonization and peri-implantitis have focused on titanium as metal, there are also reports on zirconia implants (Egawa et al. 2013; Al-Ahmad et al. 2016). These implant-centered factors may drive the compositional differences between health-diseases states and conditions. Rightly so, peri-implant infections have been described as fraternal but not as similar to periodontal infections (Robitaille et al. 2016).

## Targeted Identification of Peri-Implantitis–Associated Pathogens

Early reports that attempted to identify bacterial members associated with peri-implant infections relied on anaerobic culture-based techniques and phase contrast microscopy (Charalampakis and Belibasakis 2015). Accordingly, mainly Gram-positive cocci and nonmotile bacilli were detected in peri-implant health. Peri-implant mucositis displayed increased presence of cocci, motile bacilli, and spirochetes, whereas further Gram-negative, motile, and anaerobic species emerged in peri-implantitis. Further closed-ended molecular techniques, such as polymerase chain reaction and its variants, fluorescence in situ hybridization, or DNA-DNA checkerboard hybridization, defined a more precise list of bacteria detected in peri-implant infections, often involving common periodontopathogens. As examples, members of the “red complex” cluster comprising *Porphyromonas gingivalis, Tannerella forsythia, Treponema denticola*, and also other species from *Treponema* groups I to III and *Synergistetes* cluster A were typically associated with peri-implantitis (Shibli et al. 2008; Belibasakis et al. 2016). Overall, these early studies mostly pointed out similarities between peri-implant infections and gingivitis or chronic periodontitis. The only microbiological differences that seemed to emerge came from reports showing that peri-implant infections may occasionally be dominated by pathogens most commonly isolated from implanted medical devices, such as *Peptostreptococcus* spp. or *Staphylococcus epidermidis* and *Staphylococcus aureus* (Persson and Renvert 2014). Nonetheless, bacterial identification in the abovementioned reports relied on closed-ended molecular techniques, which entailed the preselection of a set of primers or probes and targeted bacterial identification toward specific taxa, often based on former knowledge derived from periodontitis. In this regard, there has been a “selection” bias, which technically precluded the identification of less studied or “unexpected” microbiota.

## Community-Based Microbial Pathogenesis of Peri-Implant Infections

In recent years, next-generation sequencing (NGS), that is, high-throughput DNA sequencing technologies, has become the method of choice for the taxonomic and functional characterization of the oral microbiota. NGS methods have led to a quantum leap in the number of sequence reads generated, thereby greatly improving coverage depth of analyses. Application of NGS has found unquestionable relevance for the study of oral ecosystems that encompass over 700 bacterial species, among which about 30% remained as yet uncultivated (Dewhirst et al. 2010). To date, bacterial identification most commonly relies on the sequencing of short amplicons (~400 bp) from the 16S ribosomal RNA (16S rRNA) gene that are then assigned a taxonomic identity by comparison with databases. The 16S rRNA gene comprises a combination of slowly evolving regions along with 9 fast-evolving (variable) regions, which differ among bacterial taxa and therefore become valuable targets for taxonomic assignment (Yarza et al. 2014). Amplicons spanning regions V1 to V2, V3 to V4, or V4 alone are typically targeted to yield representative community profiles that confidently reach the genus level (Wade and Prosdocimi 2020). This method has permitted researchers to comprehensively catalogue the diversity of bacteria in various oral niches (community profiling) and to relate these communities to healthy or diseased states of the host (Wade and Prosdocimi 2020). A more functional description of microbial communities can be obtained using whole-genome sequencing (Alcaraz et al. 2012), which reassembles shotgun metagenomic data to reconstruct complete bacterial metagenomes and predict the functional genetic potential within communities (Bowers et al. 2017). Thus far, 16S-based community profiling is still considered to enable the identification of the bacterial species with high resolution, more so than current shotgun metagenomic methods (Rausch et al. 2019).

Kumar et al. (2012) were the first to apply 16S rRNA gene amplicons sequencing to compare the subgingival and submucosal microbiota from periodontitis, peri-implantitis, and periodontal and peri-implant healthy sites (Kumar et al. 2012). They showed that peri-implant microbiotas harbored a significantly lower diversity and differential abundance than periodontal ones, in both health and disease. In addition, several genera, such as *Burkholderia, Anaerovorax, Anaerococcus, Aerofilium*, and *Exiguobacterium*, appeared unique to the peri-implant niche. The predominant genera in the peri-implant microbiota were *Butyrivibrio, Campylobacter, Eubacterium, Prevotella, Selenomonas, Streptococcus, Actinomyces, Leptotrichia, Propionibacterium, Peptococcus, Lactococcus*, and *Treponema*. Peri-implantitis sites were associated with lower levels of *Prevotella* and *Leptotrichia* and higher levels of *Actinomyces, Peptococcus, Campylobacter*, nonmutans *Streptococcus, Butyrivibrio*, and mutans *Streptococcus* than healthy peri-implant sites. In a subsequent study, the same group refined their approach to examine peri-implant and periodontal microbiotas from adjacent sites (Dabdoub et al. 2013). Consistently, the peri-implant microbiota demonstrated significantly lower diversity compared to periodontal sites, and distinct bacterial lineages were associated with health and disease in each ecosystem. This also was the first NGS report to detect staphylococci (*Staphylococcus pettenkoferi* and *Staphylococcus hominis*) at significantly higher abundances in peri-implantitis than periodontitis microbial communities. Interestingly, 85% of individuals shared less than 8% of abundant species between pairs of adjacent peri-implant and periodontal sites.

Distinct bacterial communities between peri-implantitis and periodontitis have also been observed in another 16S rRNA-based study (Maruyama et al. 2014), with *Prevotella nigrescens* presenting at significantly higher abundance in peri-implantitis, whereas *Peptostreptococcaceae* sp. and *Desulfomicrobium orale* were significantly higher in periodontitis. An as-yet-uncultivated *Treponema* sp. HMT-257 was uniquely associated with the severity of peri-implantitis, correlating markedly with radiographic bone loss, PPD, and suppuration.

In an effort to better understand the progressive shifts that occur within microbial communities during the establishment of a peri-implant infection, Zheng et al. (2015) characterized stage-wise the microbiota from healthy peri-implant, peri-implant mucositis, and peri-implantitis sites. A gradual increase in microbial diversity from health to peri-implant mucositis and then to peri-implantitis was observed. Peri-implant mucositis, but not peri-implantitis, was significantly associated with increased abundances of “classic” periodontal pathogens, including *P. gingivalis, T. forsythia*, and *Prevotella intermedia*. The microbiota from peri-implantitis also harbored differentially abundant communities, compared to earlier clinical stages, with a marked enrichment of several members of the *Eubacterium* spp.

Tsigarida et al. (2015) adopted a similar sequencing approach to examine microbial shifts of submucosal biofilms obtained from healthy peri-implant, peri-implant mucositis, and peri-implantitis sites, while also evaluating smoking as a factor of environmental influence. At healthy sites, smokers exhibited lower bacterial diversity and increased abundances of known disease-associated species compared to nonsmokers. In smokers, the progression from health to peri-implant mucositis was accompanied by loss of several known health-associated species, consequently decreasing the bacterial diversity. In contrast, the same clinical progression in nonsmokers (from health to peri-implant mucositis) followed a primary ecological succession, whereby the newly acquired species do not replace the pioneer taxa, with a consequent increase in the microbial community diversity. Interestingly, despite significant changes between health and peri-implant mucositis, further changes from peri-implant mucositis to peri-implantitis were not significant.

Whereas these described studies relied on 454 pyrosequencing (Roche), the technology was gradually discontinued from 2013, superseded by the Illumina platforms (Goodwin et al. 2016). Despite synthetizing slightly shorter reads (approximately 2 × 300 bp vs. 800 bp for 454), Illumina generates lower error rates and higher coverage depth (Bukin et al. 2019). Sanz-Martin et al. were the first to employ MiSeq Illumina technology, still the most widely used currently, to sequence 16S rRNA gene amplicons from healthy peri-implant and peri-implantitis sites (Sanz-Martin et al. 2017). Their results identified a differentially abundant microbiota between healthy and peri-implantitis sites at all taxonomic levels. Specifically, peri-implantitis communities were enriched with the phyla *Bacteroidetes, Spirochetes*, and *Synergistetes*, whereas *Actinobacteria* prevailed at healthy sites. At lower taxonomic levels, acknowledged periodontal pathogens, such as *P. gingivalis, T. forsythia*, and *T. denticola* were more abundant in peri-implantitis sites, along with some less well-characterized taxa, including *Peptostreptococcaceae, Desulfobulbus, Treponema maltophilum, Eubacterium saphenum, Filifactor alocis, Freitbacterium fastidiosum*, and *Fretibacterium* HMT 360. In contrast, *Veillonella dispar, Rothia dentocariosa*, and *Streptococcus sanguinis* displayed significantly higher abundances at healthy sites.

An NGS study that analyzed peri-implant crevicular fluid (PICF) rather than submucosal biofilms also identified differentially abundant bacterial communities between peri-implant health and disease. Increased abundances of genera *Vibrio, Campylobacter*, and *Granulicatella* were observed in health, whereas *Acinetobacter, Micrococcus*, and *Moraxella* were enriched in peri-implantitis (Gao et al. 2018).

Another study examined a cohort of patients previously treated for aggressive periodontitis and aimed to compare the microbiotas originating from a multitude of different sites: healthy peri-implants, peri-implant mucositis, peri-implantitis, periodontal healthy sites from implant patients, successfully treated aggressive periodontitis sites, and yet-active aggressive periodontitis pockets (Sousa et al. 2017). Significant differences in the distribution of microbiota were identified between peri-implant and periodontal niches, as well as between states of health and disease. Microbial diversity was more evident in periodontal sites and highest at sites of active periodontitis. One notable finding was the identification of genera unique to peri-implant, as compared to periodontal sites: *Filifactor, Mogibacterium, Propionibacterium, Acinetobacter, Staphylococcus, Paludibacter*, and *Bradyrhizobium*. It is known that *Filifactor* is commonly associated with chronic periodontal lesions (Wei et al. 2019), and hence this finding may be somewhat unexpected. Nevertheless, it is relevant to mention that only aggressive periodontitis patients were included in this study. Also, *Paludibacter* is commonly present in swamp rice cultures, and *Bradyrhizobium* is a nitrogen-fixing symbiote associated with plant rhizomes (Ueki et al. 2006; Aserse et al. 2017). It is worth mentioning that although the presence of DNA from soil bacteria was previously shown to account for potential kits, regents, or paper points contaminations (van der Horst et al. 2013; Laurence et al. 2014; Kim et al. 2017), the discussed study reports testing negative control samples to allow the identification and exclusion of potential contaminant reads (Sousa et al. 2017). Although uncommon, the presence of soil bacteria in the oral cavity may be explained by geographical or dietary factors (Ueki et al. 2006).

To better understand why individuals with a history of chronic periodontitis are more prone to develop peri-implantitis, Apatzidou et al. (2017) examined the microbiota compositions of peri-implantitis sites and nonadjacent healthy periodontal sites in patients with a controlled periodontal status. Healthy periodontal sites exhibited a more diverse microbiota and were associated with increased abundances of the genera *Actinobacillus* and *Streptococcus.* In contrast, *Prevotella* spp. and *Porphyromonas* spp. were most discriminative of peri-implantitis.

Taken together, these 16S-based reports have undeniably expanded the catalogue of bacterial taxa identified in peri-implant infections, as compared to previous studies relying on targeted identification approaches. Perhaps not unexpectedly, microbial communities in peri-implantitis exhibited differential abundances as compared to peri-implant health or periodontitis. Only 2 reports did not identify significant differences in microbial composition or reported interindividual variations to outweigh differences between peri-implant and periodontal sites (Schaumann et al. 2014; Yu et al. 2019). Such heterogeneity between NGS studies has been previously reported in systematic reviews, imputed to inherent variations in clinical classification, inclusion/exclusion criteria, disease progression, sampling technique, or, more evidently, sequencing technology or analysis (Rakic et al. 2016; Sahrmann et al. 2020).

**Figure 1.**
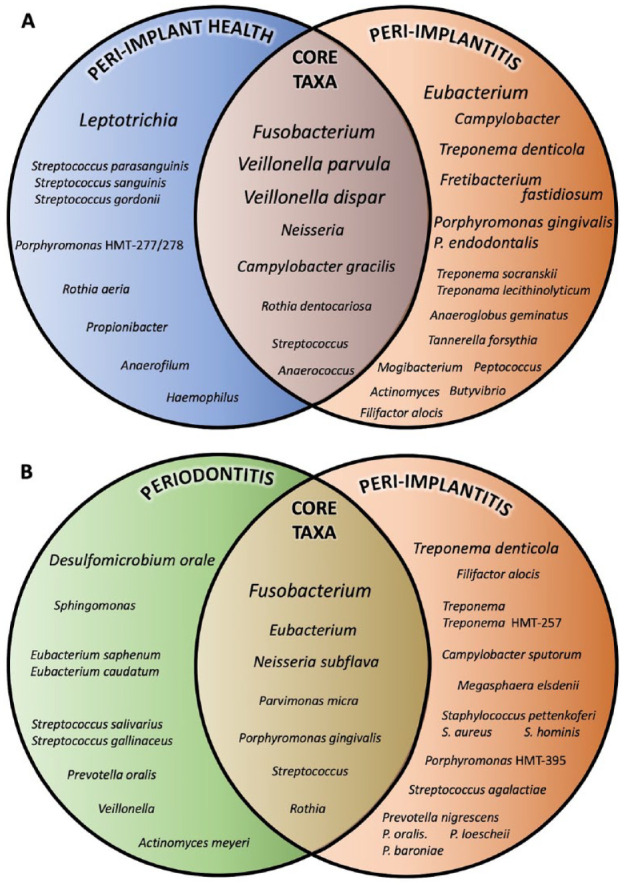
Model of characteristic and core microbiota associated with peri-implantitis. The Venn diagrams attempt a summative qualitative illustration of the characteristic taxa from the microbiota of healthy peri-implant, peri-implantitis, and periodontitis sites. Only taxa identified as significantly more abundant in each condition are represented, as reported in each individual study. (**A**) The microbiota from healthy implants and peri-implantitis are illustrated based on Kumar et al. (2012), Tsigarida et al. (2015), Zheng et al. (2015), Sanz-Martin et al. (2017), and Yu et al. (2019). (**B**) The microbiota from periodontitis and peri-implantitis sites are illustrated based on Kumar et al. (2012), Dabdoub et al. (2013), Maruyama et al. (2014), and Yu et al. (2019). Bacterial taxa are reported at the genus level or lower. The increase in font size depicts the frequency of identification among publications. Of note, criteria of taxonomic identification and statistical significance may vary among studies.

**Figure 2.**
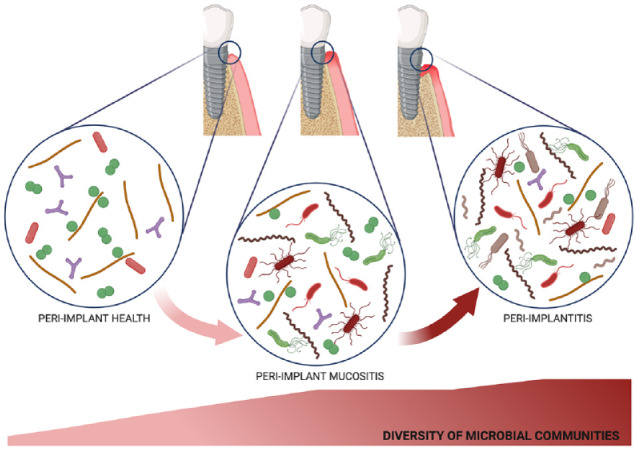
Diversity of submucosal microbial communities during the course of peri-implant infections. The scheme illustrates the increase in microbial diversity observed during the transition from peri-implant health to peri-implant mucositis and then to peri-implantitis. This figure was designed using the web interface BioRender.com.

Nonetheless, one may distinguish a collective consensus that suggests peri-implant and periodontal sites to behave as distinct ecosystems that differently shape the quantitative and qualitative composition of their residing microbiota, with limited influence from nearby niches. Peri-implant sites harbored a less diverse microbiota than periodontal sites in both health and disease. Yet, the peri-implant microbiota was shown to gradually gain complexity as the infection progressed toward peri-implant mucositis and peri-implantitis. Few bacterial taxa, such as staphylococci, frequently appeared to be distinctive of the peri-implant niche. Figure 1 provides an illustrative overview of the representative bacterial taxa that have been found to be differentially abundant between peri-implant health and peri-implantitis, as well as between peri-implantitis and periodontitis. Core taxa that are concomitantly found between these conditions are also illustrated. Peri-implant mucositis appears to exert a pivotal role in the progression of the infection, often exhibiting increased abundances of periodontal pathogens that may create a “high-at-risk-for-harm” microbiota (Fig. 2).

However, microbial differences between peri-implant conditions and periodontitis could not be attributed to the unequivocal presence or absence of specific taxa, which would assume a causal relationship. Rather, the differences were reflected in taxonomic shifts toward enriched relative abundances of entire bacterial communities.

These observations bring to notice some limitations resulting from short 16S rRNA gene amplicons, as required by most frequently employed second-generation sequencing technologies (formerly pyrosequencing or Illumina). As examples, the choice of the variable regions to sequence may introduce biases, as not all regions display comparable ability to distinguish between taxa, and taxonomic resolution is limited to the species level (Yarza et al. 2014; Wade and Prosdocimi 2020). Although recent advances in long read technologies, such as the PacBio Single Molecule, Real Time (SMRT) sequencing (Schloss et al. 2016; Callahan et al. 2019), are currently optimizing the yield of 16S approaches, more finite differences between peri-implant conditions are likely to arise only at the strain level. Besides, different strains of the same species may display significantly different virulence traits, necessitating more functional approaches for their characterization. Whereas comprehensive phylogenetic characterization of the taxa associated with peri-implant infections is an indispensable step, full elucidation of the pathological processes ultimately relies on the functional determination of their microbial pathogenicity.

## Functionality-Based Microbial Pathogenicity of Peri-Implant Infections

Although few studies have investigated the transcriptome of human cells during the course of peri-implant infections (Zhang et al. 2017; Cho et al. 2020), to the best of our knowledge, thus far, only one has addressed the corresponding microbiota. Specifically, Shiba et al. (2016) employed a genome-wide metatranscriptomic analysis (RNA-seq) on peri-implantitis and periodontitis biofilm samples to better understand the ongoing functional aspects within microbial communities. In this approach, total RNA is extracted from microbial communities and commonly directly reverse-transcribed to a complementary DNA library to be sequenced. A near-full-length 16S rRNA sequence library was first reconstructed in order to assign taxonomic identifications to bacterial communities. A differentially abundant microbial composition was confirmed between peri-implantitis and periodontitis sites. Further function-based assignment of messenger RNA (mRNA) sequences, especially focusing on putative virulence genes, identified similar functional profiles, suggesting that peri-implantitis and periodontitis are associated with similar virulence factors. To further assess whether differences in virulence profiles could emerge between healthy and diseased conditions, the authors compared their data with reads from healthy periodontal sites retrieved from another RNA-seq experiment deposited at the Human Oral Microbiome Database (Duran-Pinedo et al. 2014). This latter comparison revealed distinct virulence profiles between healthy and diseased conditions. Interaction networks appeared more complex in peri-implantitis and were characterized by significant associations between some species, which were not observed in periodontitis. Although based on a single study, these metatranscriptomic data illustrate that peri-implant infections are ultimately driven by the microbial pathogenicity of the associated bacterial communities, whereas their functional and virulence profiles are poorly reflected in their taxonomic profiles.

## Concluding Comments and Perspectives

The advent of NGS approaches to characterize the peri-implant microbiota has drastically improved our appreciation of the diversity and ecology of the bacterial communities. Peri-implant sites are distinct ecological niches, characterized by lower diversities than periodontal niches, yet harboring a differently abundant microbiota in both health and disease. These community surveys further indicated that health and disease situations were associated with compositional shifts within communities rather than the presence of specific pathogenic taxa. As examples, *Leptotrichia* spp. and *Eubacterium* spp. appear to be differentially abundant in peri-implant health and peri-implantitis, respectively, whereas *Fusobacterium* spp. and *Veillonella* spp. comprise part of their shared core microbiome (Fig. 1).

Functional and virulence differences between strains of the same species, expressed as altered transcriptional profiles, may directly potentiate the pathogenicity of the entire community. This latter field is unexplored in the context of peri-implant infections and is worth researching more extensively. It is now high-time that researching the microbiology of peri-implantitis and its differences with periodontitis serves more than the scientific curiosity of their comparative etiologies. Rather, efforts invested in deciphering the ecological triggers of functional pathogenicity might prove more beneficial to build improved strategies for risk assessment, prevention, diagnosis, or supportive therapy when required. Since metatranscriptomic pathways specific to peri-implantitis have been identified (Shiba et al. 2016), could RNA-seq or NGS profiling of submucosal biofilms or saliva be adequate to identify incipient dysbiosis, thus alerting for intensified maintenance or initial treatment, prior to the magnified clinical signs of the disease? Currently, such high-throughput profiling is not possible at the dental point of care. Yet, chair-side detection of selected microorganisms or their virulence factors is more realistic as there are available technological platforms for this purpose (Mitsakakis et al. 2016).

On the treatment side, could we preferentially target and block functional or metabolic pathways crucial to those taxa identified as differentially abundant in peri-implantitis or even at the earlier stage of peri-implant mucositis? Recent preliminary data in this direction indicate that growth, motility, and complement resistance in several oral *Treponema* species may be impaired by the administration of a specific oxidoreductase inhibitor (Reed et al. 2018) or that administration of a C3 inhibitor of the alternative complement pathway attenuates the progression of periodontitis (Bostanci et al. 2018). Despite the hypothetical nature of these open questions, we believe that a more finite understanding of peri-implant ecological pathways leading to the functional pathogenicity of the specialized peri-implant community is warranted and would ultimately prove beneficial for the long-term retention of dental implants.

## Author Contributions

G.N. Belibasakis, D. Manoil, contributed to conception, design, data acquisition, and interpretation, drafted and critically revised the manuscript. Both authors gave final approval and agree to be accountable for all aspects of the work.
